# Inter-individual variability in current direction for common tDCS montages

**DOI:** 10.1016/j.neuroimage.2022.119501

**Published:** 2022-10-15

**Authors:** Carys Evans, Catharina Zich, Jenny S.A. Lee, Nick Ward, Sven Bestmann

**Affiliations:** a Department for Clinical and Movement Neurosciences, UCL Queen Square Institute of Neurology, 33 Queen Square, London, WC1N 3BG, United Kingdom; b Wellcome Centre for Integrative Neuroimaging, FMRIB, Nuffield Department of Clinical Neurosciences, University of Oxford, Oxford, United Kingdom; c Wellcome Centre for Human Neuroimaging, UCL Queen Square Institute of Neurology, United Kingdom

**Keywords:** Transcranial electrical stimulation, Current flow modelling, Inter-individual variability, Brain stimulation

## Abstract

The direction of applied electric current relative to the cortical surface is a key determinant of transcranial direct current stimulation (tDCS) effects. Inter-individual differences in anatomy affect the consistency of current direction at a cortical target. However, the degree of this variability remains undetermined. Using current flow modelling (CFM), we quantified the inter-individual variability in tDCS current direction at a cortical target (left primary motor cortex, M1). Three montages targeting M1 using circular electrodes were compared: PA-tDCS directed current perpendicular to the central sulcus in a posterior-anterior direction relative to M1, ML-tDCS directed current parallel to the central sulcus in a medio-lateral direction, and conventional-tDCS applied electrodes over M1 and the contralateral forehead. In 50 healthy brain scans from the Human Connectome Project, we extracted current direction and intensity from the grey matter surface in the sulcal bank (M1_BANK_) and gyral crown (M1_CROWN_), and neighbouring primary somatosensory cortex (S1_BANK_ and S1_CROWN_). Results confirmed substantial inter-individual variability in current direction (50%–150%) across all montages. Radial inward current produced by PA-tDCS was predominantly located in M1_BANK_, whereas for conventional-tDCS it was clustered in M1_CROWN_. The difference in radial inward current in functionally distinct subregions of M1 raises the testable hypothesis that PA-tDCS and conventional-tDCS modulate cortical excitability through different mechanisms. We show that electrode locations can be used to closely approximate current direction in M1 and precentral gyrus, providing a landmark-based method for tDCS application to address the hypothesis without the need for MRI. By contrast, ML-tDCS current was more tangentially orientated, which is associated with weaker somatic polarisation. Substantial inter-individual variability in current direction likely contributes to variable neuromodulation effects reported for these protocols, emphasising the need for individualised electrode montages, including the control of current direction.

## Highlights

• Radial inward current can be delivered to different subregions of M1.
• Targeting bank versus crown may modulate excitability through different mechanisms.
• Large inter-individual variability in current direction occurs across montages.
• Electrode locations help approximate current direction across the precentral gyrus.
• Individualised control of current direction could minimise variability.

## 1 Introduction

Transcranial direct current stimulation (tDCS) is a non-invasive brain stimulation technique for modulating brain activity (Nitsche and Paulus, 2000; Nitsche and Paulus, 2011; Yavari et al., 2018; Lefaucheur et al., 2017). However, tDCS effects are often variable (Yavari et al., 2018; Hill et al., 2016; Horvath et al., 2015; Kalu et al., 2012; Wiethoff et al., 2014), limiting its efficacy. TDCS is typically applied using a fixed electrode montage and a fixed dose (e.g. 1 mA). This one-size-fits-all approach does not account for inter-individual differences in anatomy and result in variable trajectories of tDCS current between subjects (Antonenko et al., 2021; Datta et al., 2011; Li et al., 2015; Laakso et al., 2015). Using current flow models (CFM), dose-controlled tDCS can help to individualise tDCS delivery. This approach attempts to reduce variability of tDCS outcomes by maximising electric field (E-field) intensity or focality in a target region (Fernández-Corazza et al., 2020; Saturnino et al., 2019a; Dmochowski et al., 2011) and by minimising E-field variability across individuals (Caulfield et al., 2020; Evans et al., 2020). However, current direction is rarely discussed in the context of variability or dose-control (Saturnino et al., 2019b; Laakso et al., 2019; Lee et al., 2021).

**Fig. 1:**
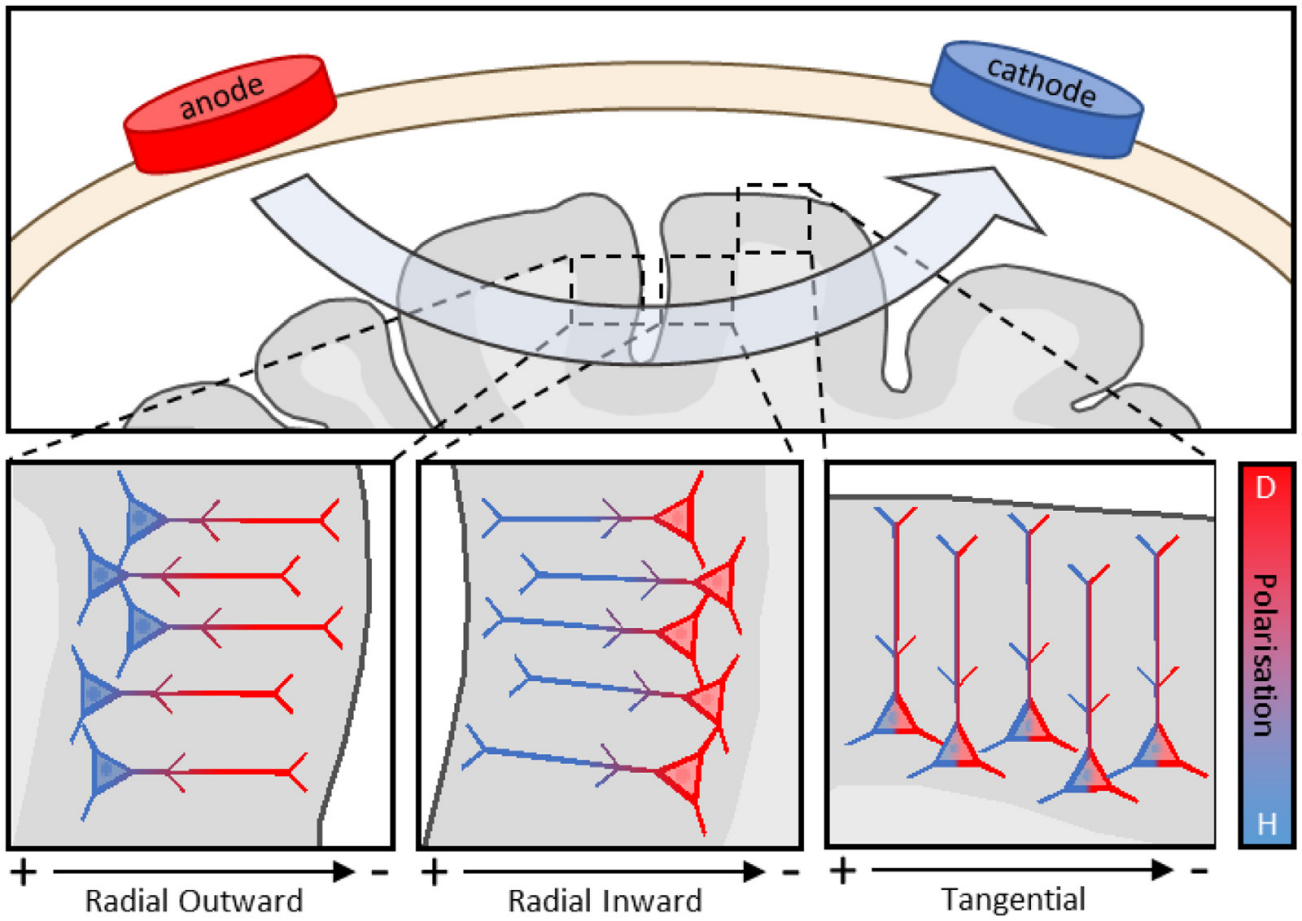
**Polarisation effects of electric current depend on the orientation of cortex**. Depolarisation (D) occurs when current flows parallel to the somatodendritic axis from dendrite to soma (radial inward), hyperpolarisation (H) when current flows soma to dendrite (radial outward), and little to no polarisation when current is orthogonal to the somatodendritic axis of the neuron (tangential).

The direction of current with respect to the orientation of the somatodendritic axes of neurons being stimulated is a primary determinant of the physiological impact of tDCS (Farahani et al., 2021; Lafon et al., 2017; Bikson et al., 2004; Rahman et al., 2013; Paulus et al., 2013; Seo and Jun, 2019; Rahman et al., 2015). Current flowing parallel to the somatodendritic axis – hereon referred to as ‘radial’ orientation – can cause somatic depolarisation or hyperpolarisation: current flowing inward from dendrite to soma causes depolarisation, whereas current flowing outward from soma to dendrite causes hyperpolarisation (Fig. 1). By contrast, current applied orthogonally to the somatodendritic axis – hereon referred to as ‘tangential’ orientation – results in little to no somatic polarisation (Farahani et al., 2021; Lafon et al., 2017; Bikson et al., 2004; Rahman et al., 2013; Reato et al., 2013).

Stimulation effects are not solely the result of somatic polarisation, and tDCS lacks the precision to exclusively target a specific population of neurons. However, the “net effect” of stimulation can be approximated by the predominant direction of current in a cortical target region (Lafon et al., 2017; Rahman et al., 2013; Radman et al., 2009). As pyramidal neurons are orientated with the long dendrites pointing towards the surface of the cortex (Radman et al., 2009; Mountcastle, 1997), the cortical surface provides a proxy for the orientation of pyramidal neurons within the primary cortical target for stimulation.

TDCS is conventionally applied by placing the anode or cathode over the target site to respectively increase or decrease excitability of the underlying neurons. However, the direction of current in the brain below an electrode is influenced by cortical folding, with morphological differences causing local fluctuations in the path of current (Rahman et al., 2013; Rawji et al., 2018; Hannah et al., 2019; Dmochowski et al., 2012). One way to mitigate this is to situate the cortical target between electrodes, resulting in greater homogeneity of current direction in the target (Rawji et al., 2018; Hannah et al., 2019; Rampersad et al., 2014; Datta et al., 2009). Rawji and colleagues (Rawji et al., 2018) demonstrated that changes to motor excitability were more consistent when tDCS electrodes were applied in a posterior-anterior (PA) orientation perpendicular to, and either side of the primary motor cortex (M1) being targeted, compared to medio-lateral (ML) electrode placement. A possible explanation for this, yet to be quantified, is that radial current becomes more consistent across the hand region of M1, situated in the posterior bank of the precentral gyrus (Lafon et al., 2017; Rahman et al., 2013; Rawji et al., 2018; Salvador et al., 2011).

Here, we assessed whether current direction in M1 differs depending on applied montage, and whether different montages produce greater radial current in different subregions of M1 (sulcal bank and gyral crown). We also assessed whether inter-individual variability in current direction differed across montages. Finally, we demonstrate how electrode positions based on landmark EEG locations can approximate the direction of current in the M1 bank and precentral gyrus, thus providing a practical solution for directing current to a cortical target in a way that reduces variance in current direction. This approach is useful where individual MRIs or expertise in current flow modelling are not available.

Current direction was quantified across the grey matter surface and in M1 when delivering fixed-intensity tDCS through three montages targeting left M1: a posterior-anterior (PA-tDCS) montage with the anode and cathode positioned anteriorly and posteriorly to the M1 hand region and current directed perpendicular to the central sulcus (Rawji et al., 2018), a medio-lateral (ML-tDCS) montage with the anode and cathode placed laterally and medially to the M1 hand area and current directed parallel to the central sulcus (Rawji et al., 2018), and a conventional montage (conventional-tDCS) with the anode over M1 and cathode over the contralateral forehead (Nitsche and Paulus, 2000; Paulus et al., 2012). We additionally compared current direction and intensity in the sulcal banks and gyral crowns of the primary motor (M1) and sensory (S1) cortices.

## 2 Materials and methods

### 2.1 Structural MRIs

Fifty T1-weighted structural MRIs of healthy adults (aged 22–35, 21 males, 29 females) were randomly selected from the Human Connectome Project (HCP) database (http://ida.loni.usc.edu/login/jsp). Subjects were scanned in a Siemens 3.0TS Connectome Skyra using a standard 32-channel head coil (0.7 mm isotropic spatial resolution, TR: 2400 ms, TE: 2.14 ms, TI: 1000 ms, flip angle: 8°, field of view: 224 × 224 mm using Siemens AutoAlign feature, iPAT: 2). An optical motion tracking system (Moire Phase Tracker, Kenticor) was used to track head movements.

The HCP is supported by the National Institute of Dental and Craniofacial Research (NIDCR), the National Institute of Mental Health (NIMH) and the National Institute of Neurological Disorders and Stroke (NINDS). HCP is the result of efforts of co-investigators from the University of Southern California, Martinos Centre for Biomedical Imaging at Massachusetts General Hospital (MGH), Washington University, and the University of Minnesota.

### 2.2 Current flow modelling

E-field modelling for tDCS was performed using Realistic vOlumetric Approach to Simulate Transcranial Electric Stimulation (ROAST) v3.0 software package (https://www.parralab.org/roast/) (Huang et al., 2019a). ROAST uses structural MRI volumes with 1mm^3^ voxel resolution to generate a 3D-rendering of E-field based on a simulated tDCS protocol. MR images are transformed into RAS space and segmented into grey matter, white matter, cerebrospinal fluid (CSF), bone, skin, and air cavities using SPM12 (http://www.fil.ion.ucl.ac.uk/spm/). ROAST automatically removes holes from segmented images (detailed in (Huang et al., 2019b; Huang et al., 2013)) before placing electrodes on the scalp using 10–10 coordinates. To generate the finite element model (FEM), ROAST creates a volumetric mesh from 3D multi-domain images using iso2mesh toolbox (http://iso2mesh.sourceforge.net/cgi-bin/index.cgi) (Fang and Boas, 2009). The FEM is then solved for current distribution using getDP FEM solver (https://getdp.info/) (Dular et al., 1998). ROAST produces E-field vectors representing current direction and intensity (V/m) at each voxel (in x-, y-, and z- dimensions). Default conductivity values were used (in S/m): grey matter: 0.276, white matter: 0.126, CSF: 1.65, bone: 0.01, skin: 0.465, air: 2.5 × 10^–14^, gel: 0.3, electrode: 5.9 × 10^7^.

### 2.3 tDCS protocol

Current flow was obtained from three user-defined bipolar electrode montages targeting the hand region of left M1 using 10–10 coordinates (Nitsche and Paulus, 2000; Paulus et al., 2013; Rawji et al., 2018; Tremblay et al., 2017): A posterior-anterior (PA) montage placed electrodes anteriorly and posteriorly to the M1 hand area, with current directed perpendicular to the central sulcus in a posterior-anterior direction (CP3: anode, FCz: cathode), a medio-lateral (ML) montage with electrodes placed medially and laterally to the M1 hand area, and current directed parallel to the central sulcus in a medio-lateral direction (CPz: anode, FC3: cathode), and a conventional montage with electrodes positioned over M1 and contralateral forehead (anode: C1, cathode: FP2). All simulations used 2 mA intensity and disc electrodes (17 mm radius, 2 mm height).

### 2.4 Grey matter surface generation

To determine current direction at the cortical surface, a grey matter surface mesh was generated using pial and white matter surface meshes taken from the HCP database. HCP extracted pial and white matter surfaces using FreeSurfer 5.1 software (http://surfer.nmr.mgh.harvard.edu/) plus customised steps to improve surface accuracy (for more detail see (Glasser et al., 2013)). Using these HCP surfaces for each subject, we first combined left and right hemisphere surfaces for pial and white matter into one surface. Surfaces were then transformed back into the original volume space by removing the central voxel to RAS offset introduced by FreeSurfer. The vertices of the pial and white matter surfaces were then averaged to create the final grey matter surface (vertices: *M* = 275,377, *SD*= 23,755 across subjects; faces: *M* = 550,747, *SD*= 47,510 across subjects) used to extract E-field vectors produced by ROAST (Fig. 2.5). Creating this surface ensured that values were extracted from grey matter and not adjacent CSF and white matter tissue when combining FreeSurfer and ROAST data.

**Fig. 2:**
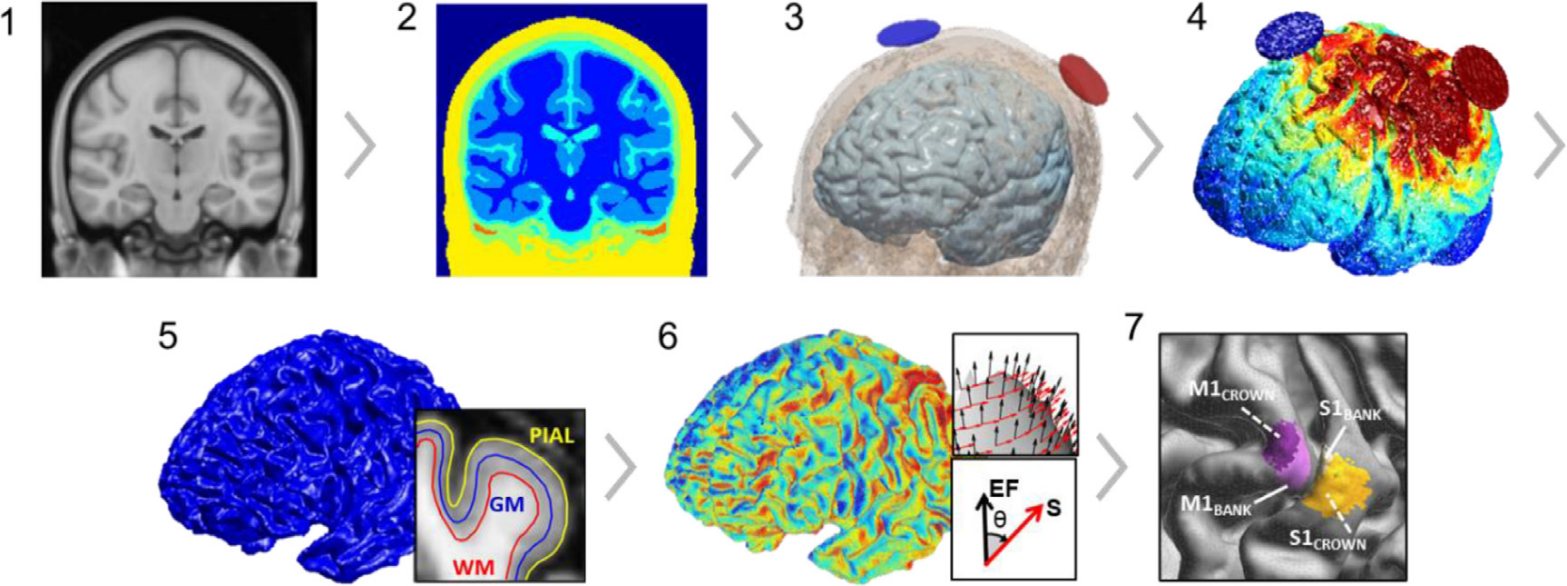
**Current flow modelling pipeline.** Steps 1–4 are automated in ROASTv3.0: using a structural MRI (1) tissues are segmented (2), electrodes are positioned (3), and the finite element model (FEM) for E-field distribution is solved (4). A grey matter (GM) surface mesh (5) is created by averaging vertices from pial and white matter (WM) surfaces generated by FreeSurfer. From the grey matter surface, surface normal vectors (S) and E-field vectors (EF) vectors are extracted, and the angular difference (degrees) between S and EF is calculated across the grey matter surface (6) and averaged within each ROI: M1_BANK_, M1_CROWN_, S1_BANK_, and S1_CROWN_ (7).

### 2.5 Regions of interest (ROI)

To quantify current direction for the three electrode montages and across individual subjects, subject-specific cortical surface ROIs were created using MATLAB (The MathWorks, Inc., Natick, MA, USA). ROIs were created on the sulcal bank and on the gyral crown of M1 and S1 (i.e., M1_BANK_ & S1_BANK_; M1_CROWN_ & S1_CROWN_).

Using the grey matter surface mesh, M1 was localised by visually identifying the “hand knob” shape anterior to the central sulcus (Dechent and Frahm, 2003; Yousry et al., 1997). At the curve of the hand knob, the centre of M1_BANK_ was marked halfway down the posterior bank of the precentral gyrus. From these coordinates, the nearest vertex of the grey matter surface mesh was identified using MATLAB's knnsearch (*k*-nearest neighbour) function. The centre of S1_BANK_ was selected by visually identifying the equivalent point opposite the centre of M1_BANK_ on the anterior bank of the postcentral gyrus. M1 and S1 crown ROIs (M1_CROWN_ & S1_CROWN_) were created by marking the centre of the gyral crown above the centre of the bank ROIs (Fig. 2).

Each ROI was generated by extending radially outward across the surface mesh from the ROI centre by five vertices; faces within these vertices were included in the ROI. Where vertices for bank and crown ROIs overlapped, each overlapping vertex was assigned to the ROI for which it had the closest geodesic distance to the ROI centre (e.g., a vertex existing in both M1_BANK_ and M1_CROWN_, but closer to the centre of M1_BANK_, was excluded from M1_CROWN_; Fig. 2).

### 2.6 Extracting E-field from grey matter surface

Using MATLAB and SPM12, E-field vectors in ROAST's model voxel space were mapped onto the grey matter surface space. To this end, the nearest E-field vectors to grey matter surface normal vectors (inner normal vector perpendicular to the surface) were identified using MATLAB's knnsearch. This subset of E-field vectors provides the estimated current direction and intensity in grey matter voxels in each ROI.

### 2.7 Calculating current direction at the cortical surface

To determine current direction relative to the grey matter surface (and by implication, the dominant orientation of pyramidal neurons), the angle (degrees) $\theta_{SEF}\;$ between surface normal vectors ($\vec{S}$) and E-field vectors ($\vec{EF}$) was calculated. Surface normal vectors provide a good proxy for the predominant orientation of pyramidal neurons due to their primary axis pointing towards the surface of the cortex. The angle between vectors ($\theta_{SEF}$) was calculated across the entire grey matter surface and within each ROI. Code for extracting E-field at cortical surface is available here: https://github.com/caryse/tdcs_currentdirection/ .

### 2.8 Using scalp electrodes to control current direction

Finally, we sought to establish whether landmark-based positioning of scalp electrodes can approximate the desired direction of current through the targeted M1 area when electrodes are placed either side of the target. This could provide a simple and accessible method for controlling current direction in the sulcal bank, which presently does not exist.

Analyses were conducted for PA-tDCS and ML-tDCS across two conditions: the location of electrodes relative to the orientation of M1_BANK_ ROIs and the ‘motor strip’ of individual subjects. These analyses determine the degree to which electrode locations provide an estimate for current direction in the cortical target and a practical solution in cases where individual scans or expertise in current flow modelling may not be available. Based on the angle (degrees) $\theta_{ELM}\;$ between electrode locations ($\vec{EL}$) and the targeted M1 area ($\vec{M}$), it is possible to adjust electrode locations to achieve the desired current direction. See Fig. 7 for concept.

Current direction as approximated by electrode location was estimated by the vector between the coordinates at the centre of each electrode (anode to cathode) for each subject. Electrode coordinates were obtained by modifying the ROAST pipeline to save the variable ‘electrode_coord’ generated through the script ‘roast.m’. The orientation of the M1_BANK_ was determined as the mean surface normal vector for M1_BANK_ ROI for each subject. To determine the dominant orientation of the ‘motor strip’, we used the vector between coordinates at each end of the precentral gyrus (medial to lateral). Motor strip coordinates were visually identified using the grey matter surface mesh as the most medial point on the crown of the precentral gyrus before the longitudinal fissure, and most lateral point on the crown before the sylvian fissure.

To maintain consistency with data obtained using surface normal vectors, the vector orthogonal (posterior to anterior) to the motor strip vector was used in angle calculations so that zero degrees denotes absolute radial-inward current, 90° is absolute tangential, and 180° absolute radial-outward. Ordinarily the motor strip vector would suffice.

### 2.9 Data analysis

Statistical analyses of current direction and intensity were carried out using R-v4.0.3 in RStudio v1.3.1093. Alpha level was 0.05 and a Bonferroni correction was applied for post-hoc multiple comparisons.

Using the mean angle ($\theta_{SEF}$) within each ROI, a linear mixed-effects model assessed differences in current direction depending on Montage (PA/ML/Conventional), Gyrus (M1/S1), ROI (sulcal Bank/gyral Crown) and associated interactions Montage x Gyrus, Montage x ROI, and Gyrus x ROI. Subject was included as the random effect on intercepts. Post-hoc pairwise comparisons explored main effects and interactions observed in the linear model. In addition, we compared E-field intensity, using the same linear mixed-effects model and post-hoc comparisons, but with mean E-field intensity (V/m) as the dependant variable.

Finally, Pearson correlations assessed the relationship between current direction and E-field intensity for each condition. Correlations also examined whether there was good correspondence between current direction in the cortical target M1_BANK_ approximated by current flow models and current direction approximated by electrode locations.

### 2.10 Comparison of volume- and surface- based modelling pipelines

As the current study uses volume-based data, which is projected onto a grey matter surface, we compared our results with surface-based models (SimNIBS v3.2) for three exemplary subjects (1, 25, 29). Previous work has shown differences in predicted fields between ROAST and SimNIBS due to loss of anatomical detail that occurs when converting volumetric data into surfaces (Huang et al., 2019a). Both the modified ROAST and SimNIBS pipelines perform volumetric to surface transformations, however, our pipeline completes the transformation as a final step whereas SimNIBS transforms data prior to mesh generation.

The equivalent ‘E_angle’ data produced by SimNIBS reflects the angle between current direction and surface normal of a “central” cortical layer (between pial and white matter surfaces) and is thus conceptually comparable to the current direction estimates obtained in our analyses. The lack of ground truth data, render interpretation of direct quantitative comparisons between these estimates difficult, and we opted instead for qualitative assessment of these data.

We observed that estimates of current direction for both pipelines were qualitatively highly congruent, suggesting our pipeline produces comparable estimates of current direction. Appendix Fig. A shows current direction results in the pre- and post- central gyri for both the modified ROAST and SimNIBS pipelines.

## 3 Results

### 3.1 Current direction across the cortical surface varies between montages

**Fig. 3:**
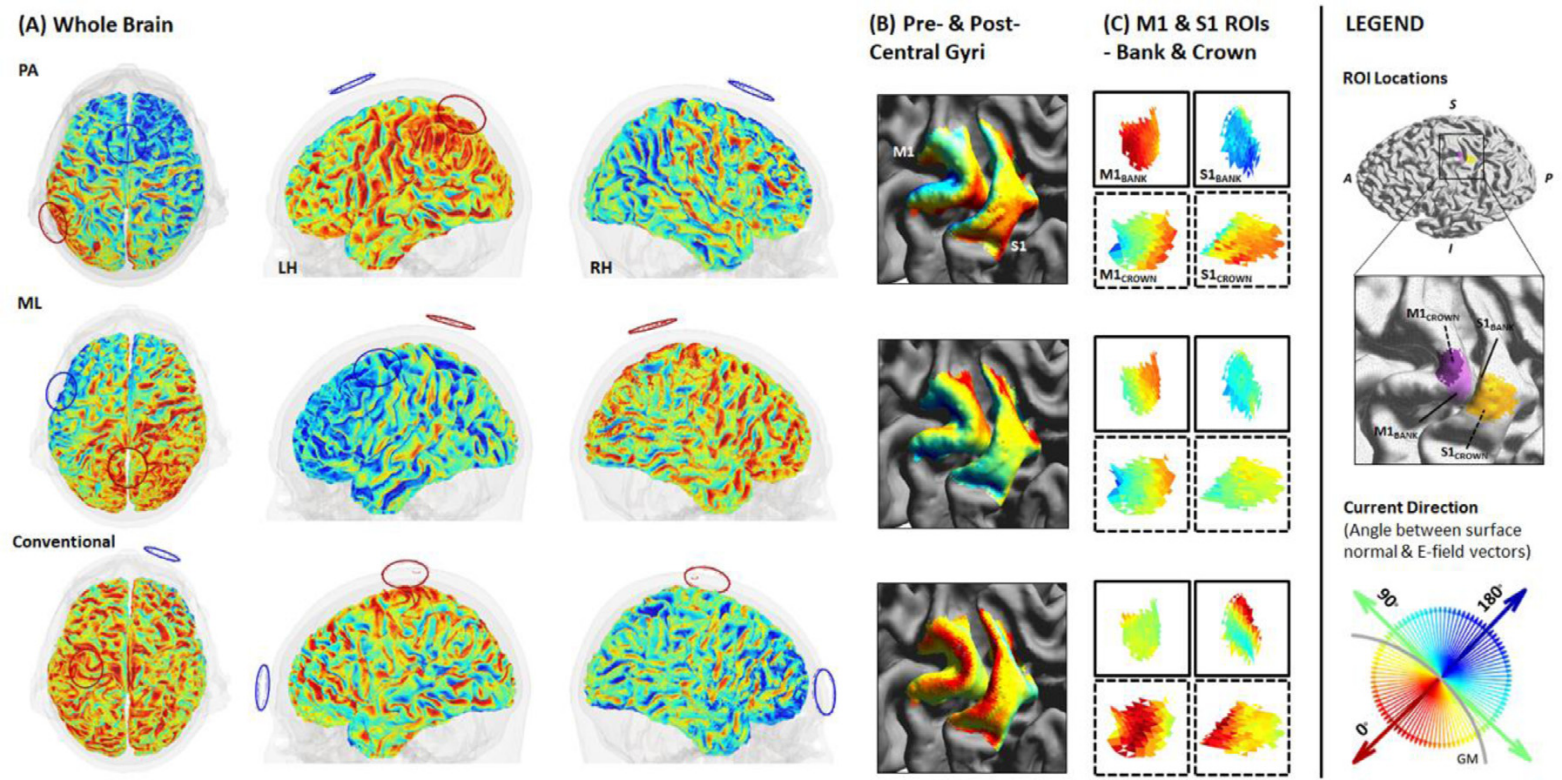
**Current direction for each electrode montage in a single subject.** Current direction (angle in degrees between surface normal (S) and E-field (EF) vectors) is depicted across the whole brain (A), pre- and post- central gyri (B) and individual M1 and S1 bank and crown ROIs (C). Bank and crown data are indicated using solid and dashed boxes, respectively. ROI locations depicted in purple (M1_BANK_ & M1_CROWN_) and yellow (S1_BANK_ & S1_CROWN_). Note opposing radial inward and outward current in M1_BANK_ and S1_BANK_ when applying a posterior-anterior montage (PA-tDCS).Conventional-tDCS produces relatively consistent radial inward current in M1_CROWN_ and S1_CROWN_, whereas a medio-lateral montage (ML-tDCS) produces tangential current across all ROIs.

**Fig. 4:**
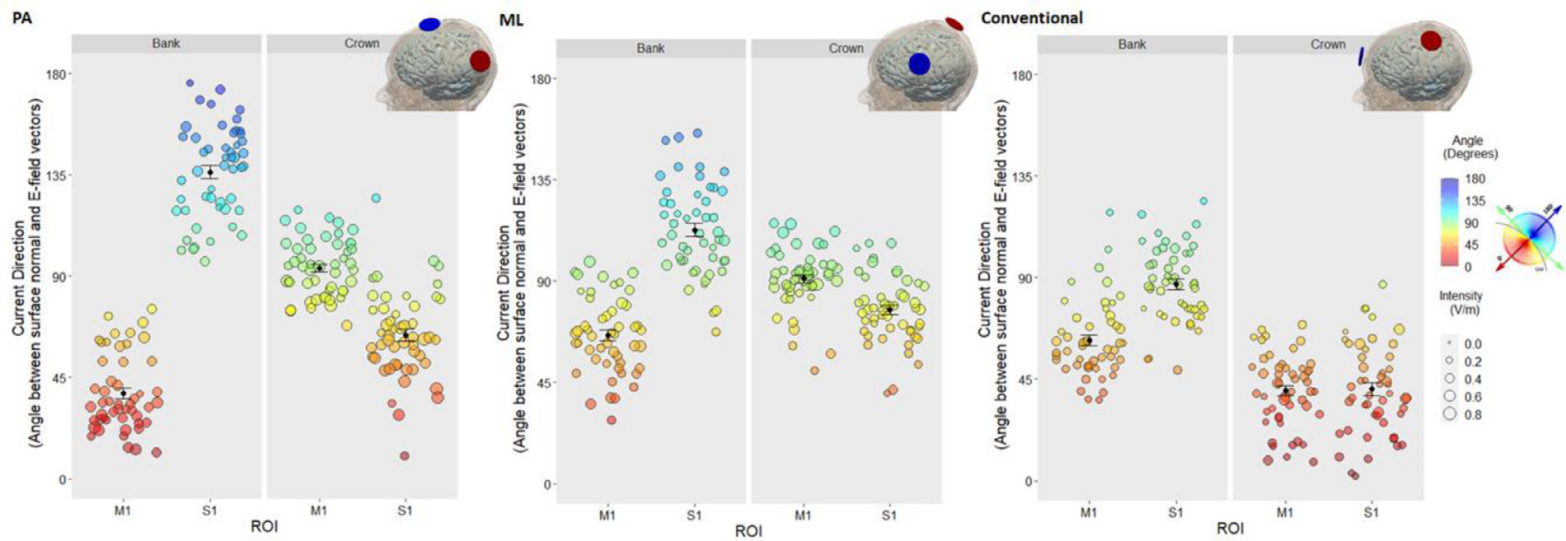
**Inter-individual variability in current direction for different montage and cortical locations.** Mean angle (degrees) between surface normal and E-field vectors of each subject for each Montage (PA, ML, Conventional), gyrus (M1/S1) and ROI (Bank/Crown). Data points represent individual subjects, with the radius denoting E-field intensity (V/m), and colour and y-axis denoting the angle between surface normal and current direction. Black data points and error bars: mean and standard error across subjects. Note the extensive inter-individual variability in current direction regardless of montage: Posterior-anterior (PA), medio-lateral (ML), and conventional.

First, we quantified current direction (angle in degrees) across the grey matter surface for each electrode montage. Across all montages, radial inward current (red colours in Figs. 3, 4 and 6) was most prominent in the gyral crowns underneath the anode, and radial outward current (blue colours in Figs. 3, 4 and 6) underneath the cathode. Beyond that, the pattern of current flow varied substantially between electrode montages (see Fig. 3A for example subject).

When applying PA-tDCS, a striped pattern of inward and outward radial current was observed, alternating between posterior and anterior banks of sulci located between the electrodes (Fig. 3A top row). This was most marked in a left-posterior to right-anterior pattern, in line with the anode and cathode locations for this montage. On the gyral crowns, current flow was predominantly tangential (green colours in Figs. 3, 4 and 6) relative to the cortical surface, except in regions underneath the anode and cathode.

ML-tDCS (Fig. 3A middle row) produced similar inward and outward radial current in opposing sulci, in a right-posterior to left-anterior pattern. Unlike PA-tDCS, tangential current mostly occurred across pre- and post- central sulcal banks. This is to be expected given that ML-tDCS directs current along the gyri, as opposed to perpendicularly when using PA-tDCS (Rawji et al., 2018).

Unlike PA-tDCS or ML-tDCS, inward and outward radial current for conventional-tDCS (Fig. 3A bottom row) was predominantly located in the gyral crowns. Tangential current was observed across the sulcal banks between electrodes (Fig. 3).

### 3.2 Current direction in M1 and S1 differs depending on electrode montage

Using the mean angle (degrees) between surface normal and E-field vectors within each ROI, a linear mixed-effects model quantified whether current direction differed depending on Montage (PA/ML/Conventional), Gyrus (M1/S1) and ROI (Bank/Crown). This analysis confirmed that the above mentioned patterns of current flow across the bank and crown of M1 and S1, respectively, depend on electrode montage: Montage (*F*_(2588)_ = 172.103, *p*<.001, η_p_^2^ = 0.37), Gyrus (*F*_(1588)_ = 226.259, *p*<.001, η_p_^2^ = 0.28), ROI (*F*_(1588)_ = 131.264, *p*<.001, η_p_^2^ = 0.18), Montage x Gyrus (*F*_(2588)_ = 22.413, *p*<.001, η_p_^2^ = 0.07), Montage x ROI (*F*_(2588)_ = 43.143, *p*<.001, η_p_^2^ = 0.13), Gyrus x ROI (*F*_(1588)_ = 641.616, *p*<.001, η_p_^2^ = 0.52), Gyrus x Montage x ROI (*F*_(2588)_ =118.303, *p*<.001, η_p_^2^ = 0.29). Next, we investigate current direction across montages in the M1 and S1 banks followed by M1 and S1 crowns.

### 3.3 PA-tDCS produces radial inward current in M1_BANK_, but opposing outward current in S1_BANK_

Post-hoc pairwise comparisons first determined which montage produced greater radial inward current in target area M1_BANK_ and whether similar current direction was observed in S1_BANK._

In the M1_BANK_ (mean angle in M1_BANK_ x montage), current direction was closer to radial inward when applying PA-tDCS compared to ML- (*t*_(539)_=8.184, *p*<.001) and conventional-tDCS (*t*_(539)_=7.083, *p*<.001). Comparable current direction between ML- and conventional- tDCS indicated greater tangential or near-tangential current in the M1_BANK_ (*t*_(539)_=−1.101, *p*=.814) with these montages.

In the S1_BANK_ (mean angle in S1_BANK_ x Montage), current direction differed between all montages: PA x ML (*t*_(539)_=−6.824, *p*<.001), PA x conventional (*t*_(539)_=−14.344, *p*<.001), ML x conventional (*t*_(539)_=−7.520, *p*<.001). PA-tDCS produced current closer to radial outward in the adjacent and functionally relevant S1_BANK_. ML-tDCS produced a similar but reduced pattern of current close to radial outward in this region, whereas greater tangential or near-tangential current was observed with conventional-tDCS (Table 1; Fig. 4).

Additional post-hoc pairwise comparisons (mean angle in M1_BANK_ vs S1_BANK_) confirmed that within each montage, current direction differed between M1_BANK_ and S1_BANK_: PA (*t*_(539)_=−28.631, *p*<.001), ML (*t*_(539)_=−13.622, *p*<.001), and conventional (*t*_(539)_=−7.204, *p*<.001).

These observations suggest that M1_BANK_ is best targeted with PA-tDCS, whereas ML-tDCS or conventional-tDCS may minimally target neurons in this region. The opposing current direction observed in M1_BANK_ and S1_BANK_ with a PA-tDCS montage likely leads to opposing polarisation and hence opposing modulatory effects in these regions; it is currently unknown what the net excitability effect of this antagonistic polarisation pattern would be.

### 3.5 Conventional-tDCS delivers radial inward current to both the M1_CROWN_ and S1_CROWN_

We then assessed whether a similar pattern of current direction occurred in M1_CROWN_ and S1_CROWN_ using the same post-hoc pairwise comparisons as above.

In the M1_CROWN_ (mean angle in M1_CROWN_ x Montage) we observed comparable current direction (i.e., tangential or near-tangential current) for both PA-tDCS and ML-tDCS (*t*_(539)_ = −0.770, *p* = 1.0), with current direction closer to radial inward when applying conventional-tDCS compared to PA-tDCS (*t*_(539)_ = −15.646, *p* <0.001) or ML-tDCS (*t*_(539)_ = −14.877, *p* <0.001). Table 1; Fig. 4.

**Table 1:** Current direction (angle, degrees) and intensity (V/m) approximated by current flow models (CFM) for different montages and cortical locations, for *n* = 50 subjects; current direction (angle, degrees) approximated by electrode location (EL) relative to M1_BANK_ and motor strip across subjects.

Montage	Current Direction approx. by CFM, Angle (degrees)				Intensity (V/m)				Current Direction approx. by EL, Angle (degrees)	
	M1		S1		M1		S1		Individual Subjects	
	Bank	Crown	Bank	Crown	Bank	Crown	Bank	Crown	M1_BANK_	Motor Strip
*Mean (SE)*										
PA	38.0 (2.39)	93.6 (1.77)	136.0 (2.91)	63.8 (2.73)	0.436 (0.015)	0.484 (0.016)	0.353 (0.012)	0.524 (0.021)	37.1 (1.95)	18.4 (0.49)
ML	66.1 (2.40)	91.0 (1.88)	113.0 (2.88)	77.1 (2.06)	0.409 (0.013)	0.432 (0.016)	0.338 (0.011)	0.369 (0.013)	65.2 (2.74)	73.2 (0.83)
Conventional	62.3 (2.38)	40.0 (2.24)	87.0 (2.38)	40.6 (2.82)	0.266 (0.01)	0.277 (0.015)	0.248 (0.01)	0.262 (0.014)	–	–
*Range*										
PA	11.8–75.4	68.1–119.0	96.1–176.0	10.0–125.0	0.218–0.785	0.234- 0.696	0.163–0.526	0.244–0.874	10.7–69.6	8.65–25.4
ML	28.2–98.7	50.2–116.0	67.4–156.0	40.1–107.0	0.209–0.606	0.184–0.714	0.169–0.490	0.181–0.582	26.6–103.0	62.9–86.7
Conventional	35.7–119.0	8.27–69.1	48.9–124.0	1.83–87.1	0.129–0.431	0.122–0.771	0.103–0.376	0.105–0.529	–	–

*Note:* 0° = absolute radial-inward; 90° = absolute tangential; 180° = absolute radial-outward current.

In the S1_CROWN_ (mean angle in S1_CROWN_ x Montage) current direction differed between all montages: PA x ML (*t*_(539)_=3.889, *p*<.001), PA x conventional (*t*_(539)_= −6.752, *p*<.001), ML x conventional (*t*_(539)_= −10.641, *p*<.001). As above, PA-tDCS and ML-tDCS produced greater tangential or near-tangential current S1_CROWN_ compared to current closer to radial inward direction when applying conventional-tDCS.

Comparing current direction between M1_CROWN_ and S1_CROWN_ (mean angle in M1_CROWN_ vs S1_CROWN_) within each montage indicated that conventional-tDCS produced comparable current direction in M1_CROWN_ and S1_CROWN_ (*t*_(539)_= −0.180, *p*=.857), whereas current direction differed with PA-tDCS (*t*_(539)_= 8.714, *p*<.001, more radial inward current in S1_CROWN_ than M1_CROWN_) and ML-tDCS (*t*_(539)_= 4.056, *p*<.001, more radial inward current in S1_CROWN_ than M1_CROWN_).

These results suggest that targeting M1_CROWN_ is best achieved using a conventional tDCS montage. Conventional-tDCS does not result in opposing current direction in the adjacent S1_CROWN_, as might be expected given the size of the electrodes positioned directly over this location.

### 3.6 PA-tDCS produces highest current intensities in both the bank and crown of M1 and S1

An additional linear mixed-effects model confirmed that mean E-field intensity (V/m) in each ROI differed depending on Montage, Gyrus, and ROI: Montage (*F*_(2539)_ = 545.068, *p*<.001, η_p_^2^ = 0.67), Gyrus (*F*_(1539)_ = 56.628, *p*<.001, η_p_^2^ = 0.10), ROI (*F*_(1539)_ = 112.386, *p*<.001, η_p_^2^ = 0.17), Montage x Gyrus (*F*_(2539)_ = 11.711, *p*<.001, η_p_^2^ = 0.04), Montage x ROI (*F*_(2539)_ = 41.387, *p*<.001, η_p_^2^ = 0.13), Gyrus x ROI (*F*_(1539)_ =22.599, *p*<.001, η_p_^2^ = 0.04), Gyrus x Montage x ROI (*F*_(2539)_ =17.398, *p*<.001, η_p_^2^ = 0.06).

**Fig. 5:**
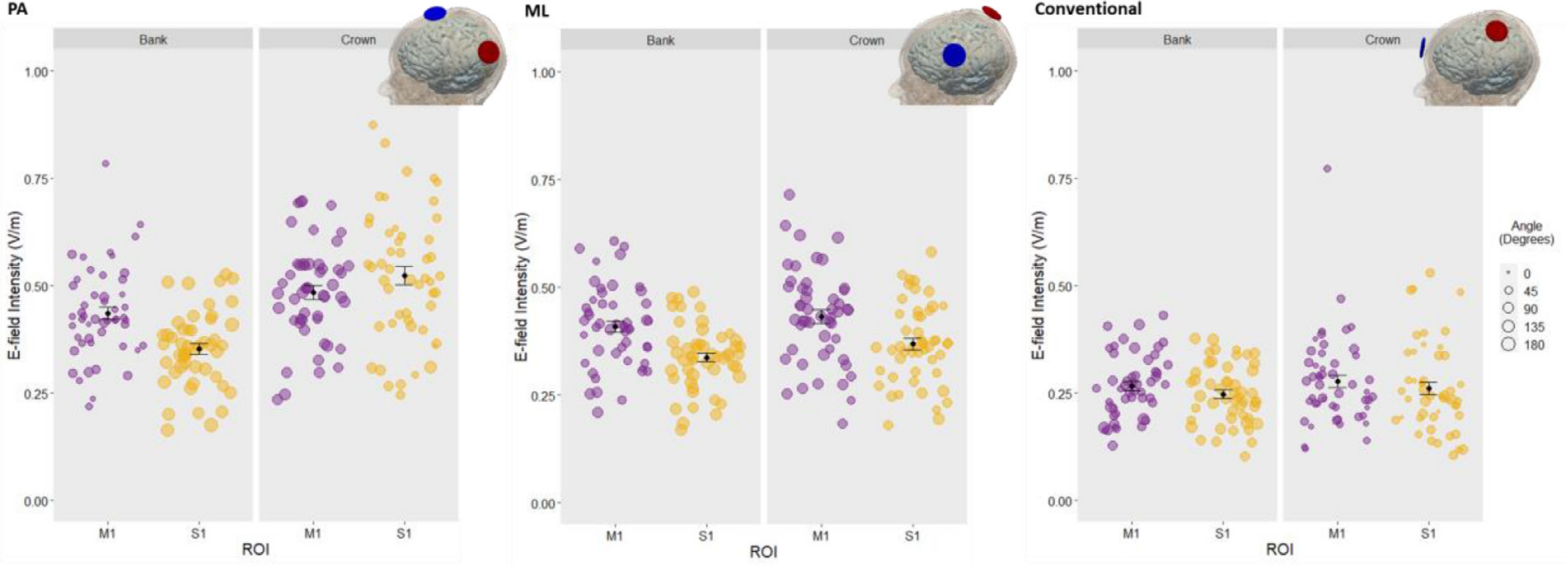
**Inter-individual variability in mean E-field intensity (V/m) for different montage and cortical locations.** Data points represent individual subjects; their size denotes angle (degrees) between surface normal and E-field vectors. Black data points and error bars: mean and standard error across subjects. Note highest intensities in both bank and crown ROIs were observed with PA-tDCS and lowest with conventional-tDCS. All montages show high inter-individual variability in intensity.

In the M1_BANK_ (mean intensity in M1_BANK_ x Montage), significantly higher current intensities were produced by PA-tDCS (*t*_(539)_= 14.780, *p*<.001) and ML-tDCS (*t*_(539)_= 12.425, *p*<.001) when compared to conventional-tDCS. Intensities were comparable between PA-tDCS and ML-tDCS (*t*_(539)_=2.356, *p*=.057). Table 1; Fig. 5. Similarly, in the S1_BANK_ (mean intensity in S1_BANK_ x Montage), PA-tDCS (*t*_(539)_= 9.222, *p*<.001) and ML-tDCS (*t*_(539)_= 7.850, *p*<.001) produced higher intensities compared to conventional-tDCS. Intensities between PA-tDCS and ML-tDCS were comparable (*t*_(539)_= 1.372, *p*=.512).

In the M1_CROWN,_ PA-tDCS produced higher current intensities than ML-tDCS (*t*_(539)_= 4.529, *p*<.001) and conventional-tDCS (*t*_(539)_= 18.001, *p*<.001). Intensities were lowest when applying conventional-tDCS compared to ML-tDCS (*t*_(539)_= 13.473, *p*<.001). In the S1_CROWN_, PA-tDCS also produced higher intensities than both ML-tDCS (*t*_(539)_= 13.532, *p*<.001) and conventional-tDCS (*t*_(539)_=22.875, *p*<.001), and intensities were again lowest when applying conventional-tDCS compared to ML-tDCS (*t*_(539)_= 9.344, *p*<.001).

Comparing intensities in M1_BANK_ and M1_CROWN_ (mean intensity in M1_BANK_ vs M1_CROWN_), intensities were higher in M1_CROWN_ for PA-tDCS (*t*_(539)_= −4.185, *p*<.001) and ML-tDCS (*t*_(539)_= −2.012, *p*=.045), but comparable between M1_BANK_ vs M1_CROWN_ for conventional-tDCS (*t*_(539)_= −0.964, *p*=.335). These patterns were also observed when comparing S1_BANK_ and S1_CROWN_: PA: *t*_(539)_= −14.873, *p*<.001, ML: *t*_(539)_= −2.713, *p*=.007, conventional: *t*_(539)_= −1.220, *p*=.223.

PA-tDCS produces the highest current intensities across all ROIs compared to ML-tDCS and conventional-tDCS. Notably, PA-tDCS produced almost double current intensities in target M1_BANK_ and M1_CROWN_ compared to a conventional-tDCS. Compared to ML-tDCS, PA-tDCS produced higher intensities in M1_CROWN_ but comparable intensities in M1_BANK_. Nevertheless, regardless of montage, high inter-individual variability in E-field intensity was observed: intensities in M1 varied by ∼100% with a given montage (Table 1).

### 3.7 High inter-individual variability in current direction regardless of electrode montage

Despite clear differences in the direction of current between three montages, inter-individual variance in current direction relative to the cortical surface was high for all montages. Observing the range of angles (degrees) between surface normal and E-field vectors, the difference between the lowest angle and highest angle across all conditions varied by ∼50%−150% with a given montage (Table 1).

Looking specifically at target region M1, when applying PA-tDCS current direction in M1_BANK_ ranged from 11.8° to 75.4° and between 68.1° and 119.0° in the M1_CROWN_. Similar differences were observed when applying ML-tDCS (M1_BANK_: 28.2°−98.7°; M1_CROWN_: 50.2°−116.0°) and conventional-tDCS (M1_BANK:_ 35.7°−119.0°; M1_CROWN_: 8.27°−69.1°). See Table 1 for range in M1 and S1.

**Fig. 6:**
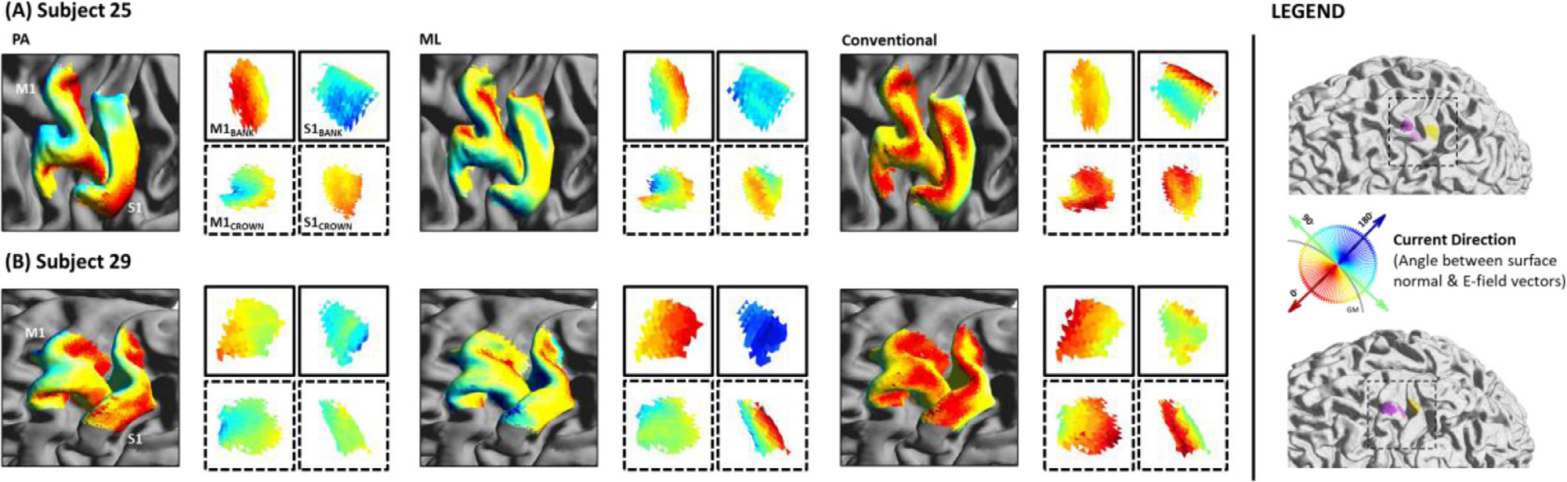
**Current direction for each electrode montage across two example subjects.** Current direction (angle in degrees between surface normal (S) and E-field (EF) vectors) is depicted across pre- and post- central gyri and individual M1 and S1 bank and crown ROIs for subject 25 (A) and 29 (B). Bank and crown data are indicated using solid and dashed boxes, respectively. ROI locations depicted in purple (M1) and yellow (S1). Note that montages producing predominantly radial inward current in either M1_BANK_ or M1_CROWN_ differ between subjects.

These results demonstrate that whilst a predominant current orientation may be observed in the cortical target area at a group-level, there is considerable variability across individual subjects. One can appreciate this variance in two example subjects (Fig. 6). Subject 25 shows a similar though less robust pattern of current direction to that observed in group-level analyses, whereas subject 29 shows a different pattern. For subject 29, ML-tDCS is preferable to PA-tDCS when targeting M1_BANK_ due to the location of the M1_BANK_ ROI relative to the direction of current.

### 3.8 Current direction and E-field intensity are largely unrelated

Pearson's correlations showed little relationship between current direction and E-field intensity for different montages and cortical target ROIs. Non-significant correlations were observed for all conditions except when applying ML-tDCS to the M1_CROWN_ (*r*_(48)_=0.333,*p*=.018) or PA-tDCS to the S1_CROWN_ (*r*_(48)_=−0.388,*p*=.005), where a weak relationship between current direction and E-field intensity was observed. Correlation results can be found in Appendix Table A.

### 3.9 Electrode location can accurately approximate current direction in the cortical target

The location of scalp electrodes provides a good approximation of current direction through the targeted M1 area, whether the target is a precise ROI (M1_BANK_) or larger cortical region (motor strip).

In the M1_BANK_, the angle (degrees) between current direction approximated by electrode location and mean surface normal vector confirmed that current direction was closer to radial inward when applying PA-tDCS and closer to tangential when applying ML-tDCS. Current direction approximated by electrode location highly correlated with the current direction estimated with CFM for both PA-tDCS (*r*_(47)_=0.913,*p*<.001) and ML-tDCS (*r*_(47)_=0.962,*p*<.001). This indicates that electrode locations provide an accurate approximation of current direction in a cortical target (see Table 1 for values).

**Fig. 7:**
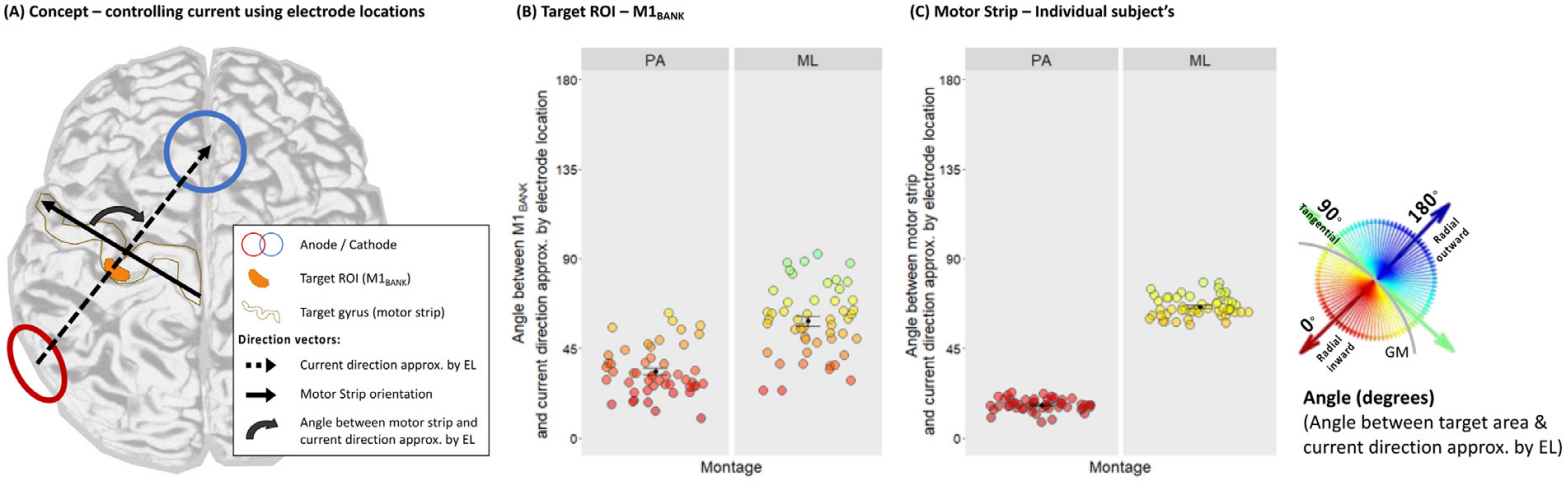
**Using electrode locations to approximate current direction in the M1_BANK_ and motor strip.** (A) Concept of current direction approximated by electrode location (EL) for PA-tDCS. The degree to which current is flowing radial-inward into the target area can be estimated by calculating the angle between EL vector (anode to cathode) and the target ROI (M1_BANK_: mean surface normal vector) or target gyrus (motor strip orientation vector: medial to lateral). (B/C) Angle (degrees) between current direction approximated by EL for PA-and ML-tDCS when targeting M1_BANK_ (B) or motor strip (C). Data points represent individual subjects with colour and y-axis denoting angle. Black datapoints and error bars: mean and standard error across subjects. Note: 0° indicates absolute radial-inward current; 90° absolute tangential, and 180° absolute radial-outward.

Using the motor strip as the targeted M1 area showed a similar distinction between PA-tDCS and ML-tDCS for individual subjects (see Fig. 7). Notably, approximating current direction across the motor strip showed less inter-individual variability. Nevertheless, using the orientation of anatomical structures such as the precentral gyrus, which can be estimated using TMS, for example, may be a useful approach for controlling current direction where individual MRIs or expertise in current flow modelling are not available.

## 4 Discussion

Using current flow modelling, we quantified current direction in target M1 region for different tDCS montages. We observed that the location of predominantly radial inward current varies with electrode montage: PA-tDCS produced largely radial inward current in the M1_BANK_, whereas conventional-tDCS produced radial inward current in the M1_CROWN_. These montages may therefore effectively target different subregions of M1 (and adjacent dorsal premotor cortex, PMd), suggesting that they may express their physiological effects through different mechanisms. Moreover, high inter-individual variability in current direction in a cortical target region likely contributes to the known variable outcomes of tDCS. We also demonstrate that current direction in a cortical target can be accurately approximated based on the location of scalp electrodes. The angle between electrode locations relative to the cortical target highly correlated with the direction of current estimated by current flow modelling. Electrode locations may therefore provide a landmark-based method for tDCS application without the need for MRI.

We observed that radial inward current delivered with PA-tDCS and conventional-tDCS was located in different regions of M1, whereas ML-tDCS produced largely tangential current in both the sulcal bank and gyral crown of M1. Using the cortical surface as a proxy for the orientation of pyramidal neurons within grey matter (Radman et al., 2009), radial inward current flowing parallel to the somatodendritic axis would likely result in somatic depolarisation, whereas tangential current flowing orthogonally to the somatodendritic axis would produce little polarisation effect (Farahani et al., 2021; Lafon et al., 2017; Bikson et al., 2004; Rahman et al., 2013; Reato et al., 2013). Our data adds insight into why tDCS effects are observed when applying PA-tDCS (Rawji et al., 2018; Hannah et al., 2019; Tremblay et al., 2017) and conventional-tDCS (Nitsche and Paulus, 2000; Paulus et al., 2013), but not when applying ML-tDCS that fails to produce radial inward current in both regions of M1 (Rawji et al., 2018).

### 4.1 Different montages may target different subregions in M1

Notably, as PA-tDCS and conventional-tDCS produced radial inward current in different regions of M1, the mechanisms by which these montages exert their net excitability changes (Nitsche and Paulus, 2000; Rawji et al., 2018) may differ. It is unknown whether targeting different neuronal populations would yield different or opposing excitability effects, though data hints that it does.

Radial inward current delivered with PA-tDCS to the sulcal bank of M1 with PA-tDCS results in suppression of motor evoked potentials (MEP) (Rawji et al., 2018). Similarly, Laakso and colleagues (Laakso et al., 2019) found that subjects with stronger normal components of E-field in this location exhibited larger decreases in MEP amplitudes than subjects with weaker normal component of E-field. By contrast, conventional-tDCS delivers radial inward current predominantly to the gyral crown of M1 and increases MEPs (Yavari et al., 2018; Nitsche and Paulus, 2001; Woods et al., 2016). In addition, current direction in S1 differs markedly between the two montages, which may indicate that differences in the observed stimulation effects read-out from motor cortex via TMS-evoked MEPs comes from the concerted interplay between the polarisation effects in M1 and S1.

For TMS, recent work suggests the primary site of activation to be at the border between dorsal premotor cortex (PMd) and M1 located in the crown of the precentral gyrus, closely reflecting M1_CROWN_ here (Salvador et al., 2011; Aberra et al., 2020; Bungert et al., 2017). The primary activation in PMd propagates downstream to intracortical circuits in the M1 hand area via transsynaptic excitation of the PMd-to-M1 hand pathway (Siebner, 2020). Reversing the TMS coil from a posterior-anterior to anterior-posterior orientation can shift the site of activation anteriorly within the M1 crown, resulting in increased MEP latencies (Aberra et al., 2020; Siebner, 2020; Dubbioso et al., 2021).

This provides a tantalizing opportunity for testing the hypothesis that tDCS can indeed preferentially modulate neural structures in the gyral crown versus bank, as suggested by our data. When applied over M1, conventional-tDCS may predominantly target neurons in M1/PMd which project to the M1 hand area, prompting an increase in MEP amplitude similar to applying TMS in a posterior-anterior orientation (Rawji et al., 2018; Siebner, 2020). By contrast, PA-tDCS may preferentially target neurons within the M1 hand area, which receive direct projections from M1/PMd. This may lead to reduced MEP amplitudes or increased MEP latencies similar to applying TMS in an anterior-posterior orientation (Rawji et al., 2018; Aberra et al., 2020; Dubbioso et al., 2021). ML-tDCS on the other hand may fail to polarize neurons responsible for MEP generation sufficiently to produce reliable changes in MEP amplitudes.

By exploiting the known latency differences in MEPs with different coil orientations (Hannah and Rothwell, 2017; D'Ostilio et al., 2016) together with the ability to target different neural elements by manipulating pulse width (Hannah and Rothwell, 2017), this hypothesis can now directly be tested. Similarly, in sensory cortex, the hypothesis for selective targeting of neural structures via control of the current direction can be directly tested using sensory stimulation (such as peripheral nerve stimulation) and sensory-evoked responses.

### 4.2 Substantial inter-individual variability in current direction irrespective of montage

Regardless of chosen montage, a large degree of inter-individual variability in current direction remains; across the sample the angle between surface normal and E-field vectors varied between 50% and 150%. Such variance likely originates from differences in the location of the cortical target relative to the standardised electrode positions. Here we identified M1 based on the “hand knob”, which is visually characterised by an omega or epsilon shape differing in prominence across individuals (Yousry et al., 1997; Caulo et al., 2007). Inter-individual differences in the shape of this region reduce the likelihood that a fixed montage will target this structure in all subjects. This can be addressed by individualised tDCS application. Moreover, such variability may be addressed by guiding individualised tDCS application based on the functional identification of the target brain region (here: M1-hand), for example, via functional magnetic resonance imaging.

Whilst interest in individualised montages has increased, their primary goal to date has been to maximise or control E-field intensities in the cortical target (Dmochowski et al., 2011; Caulfield et al., 2020; Evans et al., 2020; Laakso et al., 2019; Huang et al., 2013; Datta et al., 2012; Dmochowski et al., 2013). We demonstrate that by altering electrode montage, the mechanism by which the cortical target is modulated is effectively changed, and so are the assumptions regarding the physiological effects of stimulation. We note that tDCS optimisation is multi-factorial in nature, with inter-dependencies between E-field intensity, focality, and current direction that need considered (Lee et al., 2021). We found only a weak relationship between current direction and E-field intensity, suggesting that optimisation of tDCS ought to control both parameters (Lee et al., 2021).

### 4.3 Differences in current intensities with different electrode montages

Consistent with previous findings (Laakso et al., 2015; Caulfield et al., 2020; Evans et al., 2020; Johnstone et al., 2021) we observed a high degree (∼100%) of inter-individual variability in E-field intensity (V/m) at the cortical target location. In principle, this variance can be eliminated by adjusting tDCS delivery in each individual (see (Evans et al., 2020)).

Notably, the average intensity in both M1_BANK_ and M1_CROWN_ with PA-tDCS was almost twice as large than for conventional-tDCS. This corroborates data showing higher intensities between compared to under stimulation electrodes (Rampersad et al., 2014; Datta et al., 2012; Karabanov et al., 2019; Csifcsák et al., 2018). The presence of radial inward current and high intensities in an ‘inter-electrode’ cortical target suggests an alternative way for targeting specific cortical areas with tDCS. A corollary of this is that consideration should be given to the cortical regions located between electrodes, and how these may influence tDCS effects.

### 4.4 Landmark-based electrode locations can accurately approximate current direction

Presently, precise targeting the gyral crown or sulcal bank of M1, or any cortical target region, requires current flow models (Dmochowski et al., 2011; Saturnino et al., 2021). Here we show that a simple and practical method for that maximise radial inward current with an inter-electrode cortical target might serve as an alternative approach.

We found that current direction in a cortical target approximated by 10–10 coordinate electrode locations highly correlated with the direction of current estimated by current flow models. Electrode locations also produced similar estimates of current direction in a larger anatomical target, the motor strip, albeit capturing less inter-individual variability in current direction.

### 4.5 Limitations

Here we establish that by using the cortical surface as a proxy for neuron orientation, it is possible to quantify current direction in a cortical target using current flow modelling. Delivering more uniform current to a cortical target across subjects may improve reliability of tDCS effects. Focussing on current direction may also provide a way for generating new testable predictions about the specific neural structures targeted by tDCS.

However, some factors may affect the predictions made here. Specific to this study, HCP brain scans have undergone anonymisation and defacing steps, which will reduce the accuracy of segmentation and E-field estimation. By extension, these models treat white matter as isotropic wherein fact it is strongly anisotropic, which may lead to errors in E-field estimates of deeper brain structures (Saturnino et al., 2019a; Rampersad et al., 2014). Given that our ROIs are far from the face and our cortical targets are on the surface of grey matter, this should not significantly alter current flow estimates in our areas of interest. Further, the predictive performance of current flow models is not necessarily significantly improved when including anisotropic white matter or heterogeneous skull compartments (Huang et al., 2017). However, some gains in accuracy may be possible by using unmodified MR images or diffusion MRI to account for white matter anisotropy.

Stimulation effects are not solely driven by somatic polarisation, but also the effect of direct current on other cell compartments (dendrites, axons/terminals) and cell types (interneurons) (Lafon et al., 2017; Bikson et al., 2004). For example, tangential current polarises axons/terminals and interneurons, including corticocortical afferents (Rahman et al., 2013; Radman et al., 2009; Molaee-Ardekani et al., 2013). Accurately modelling the complex morphology of axons suggests that the net polarisation effect on neurons is caused by both radial and tangential currents (Aberra et al., 2020). To fully predict tDCS effects requires an understanding of the cumulative effects of current on all cell types and compartments and their (potentially non-linear) interplay. This necessitates realistic multi-scale modelling of both cortex and different cell types and their geometry across cortical layers, such as recently accomplished for TMS (Aberra et al., 2020). Nevertheless, whilst we better understand the effect of direct current on individual neurons through *in vitro* and neuron modelling studies (Lafon et al., 2017; Bikson et al., 2004; Rahman et al., 2013; Rahman et al., 2015; Aberra et al., 2020), our approach here provides a useful approximation for the control of current orientation at a cortical target region.

Finally, whilst optimising current direction in a precise cortical target can significantly impact tDCS outcomes (Rawji et al., 2018; Hannah et al., 2019), the overall effect of tDCS depends on stimulation effects extending beyond the cortical target (Mikkonen et al., 2019). The physiological impact of tDCS thus includes interaction of larger networks, as both radial current and high E-field intensities are observed in distal regions including contralateral M1 (Evans et al., 2020, 20; Radman et al., 2009; Datta et al., 2009; Esmaeilpour et al., 2018).

Currently, however, it is unclear how to incorporate this information in the use of current flow models and determining what to prioritise when optimizing a stimulation protocol remains a matter of debate. Both current direction (Laakso et al., 2019; Seo and Jun, 2019; Rawji et al., 2018; Hannah et al., 2019; Huang et al., 2017; Opitz et al., 2015) and E-field intensity (Antonenko et al., 2019; Foerster et al., 2019) correlate with the effects of tDCS. However, common to all CFM approaches is the question how to incorporate this information for targeting or to improve the reliability of tDCS outcomes (Dmochowski et al., 2011; Lee et al., 2021; Saturnino et al., 2021; Mikkonen et al., 2019).

## 5 Conclusion

Current flow modelling allows for quantifying inter-individual variability in current delivery of tDCS and to develop controlled and individualised tDCS approaches (Caulfield et al., 2020; Evans et al., 2020; Laakso et al., 2019; Kasten et al., 2019). Whilst attention has been given to reducing variance in E-field intensity and focality, differences in current direction across individual and protocols are rarely assessed (Saturnino et al., 2019b; Laakso et al., 2019; Rawji et al., 2018).

Using current flow modelling, the current data allows us to apply simple heuristics as to where electrodes could be placed to maximise radial inward or outward current in any cortical target in an individual, assuming this to be a key factor for the physiological effect of tDCS. What is also clear is that excitability changes are complex and partly determined by whether radial current is directed towards the sulcal bank or gyral crown of the target region. Integrating neuronal models with tDCS-induced E-fields may elucidate mechanisms to allow clear rationales when selecting electrode montages, which then require experimental validation.

## Funding

The study was funded by Brain Research UK (201617-03 / 201718-13), Dunhill Medical Trust (RPGF1810\93). The Wellcome Centre for Human Neuroimaging, UCL Queen Square Institute of Neurology, is supported by funding from the Wellcome Trust [203147/Z/16/Z]. For the purpose of open access, the author has applied a CC BY public copyright licence to any Author Accepted Manuscript version arising from this submission.

## Data availability statement

The Structural MRI data that support the findings of this study were provided by the MGH-USC Human Connectome Project. The code used to extract E-field data from these scans is available here https://github.com/caryse/tdcs_currentdirection/.

## CRediT authorship contribution statement

**Carys Evans:** Conceptualization, Methodology, Visualization, Writing – original draft. **Catharina Zich:** Methodology, Writing – review & editing. **Jenny S.A. Lee:** Methodology, Writing – review & editing. **Nick Ward:** Writing – review & editing, Funding acquisition. **Sven Bestmann:** Conceptualization, Supervision, Funding acquisition, Writing – review & editing.

## Declaration of Competing Interest

The authors declare that the research was conducted in the absence of any commercial or financial relationships that could be construed as a potential conflict of interest.

## Figures and Tables

**Table A:** Correlation between current direction (angle in degrees) and E-field intensity (V/m) for different montages and cortical locations, for *n* = 50 subjects.

Montage	Current direction (degrees) and E-field intensity (V/m)			
	M1		S1	
	Bank	Crown	Bank	Crown
PA	*r*_(48)_=−0.234,*p*=.101	*r*_(48)_=−0.267,*p*=.061	*r*_(48)_=−0.211,*p*=.141	*r*_(48)_=−0.388,*p*=.005**
ML	*r*_(48)_=0.068,*p*=.639	*r*_(48)_=0.333,*p*=.018*	*r*_(48)_=−0.142,*p*=.324	*r*_(48)_=0.173,*p*=.230
Conventional	*r*_(48)_=0.029,*p*=.838	*r*_(48)_=0.105,*p*=.467	*r*_(48)_=0.084,*p*=.561	*r*_(48)_=−0.060,*p*=.678

*Note:* *p < .05*;* **p < .01.

**Fig. A:**
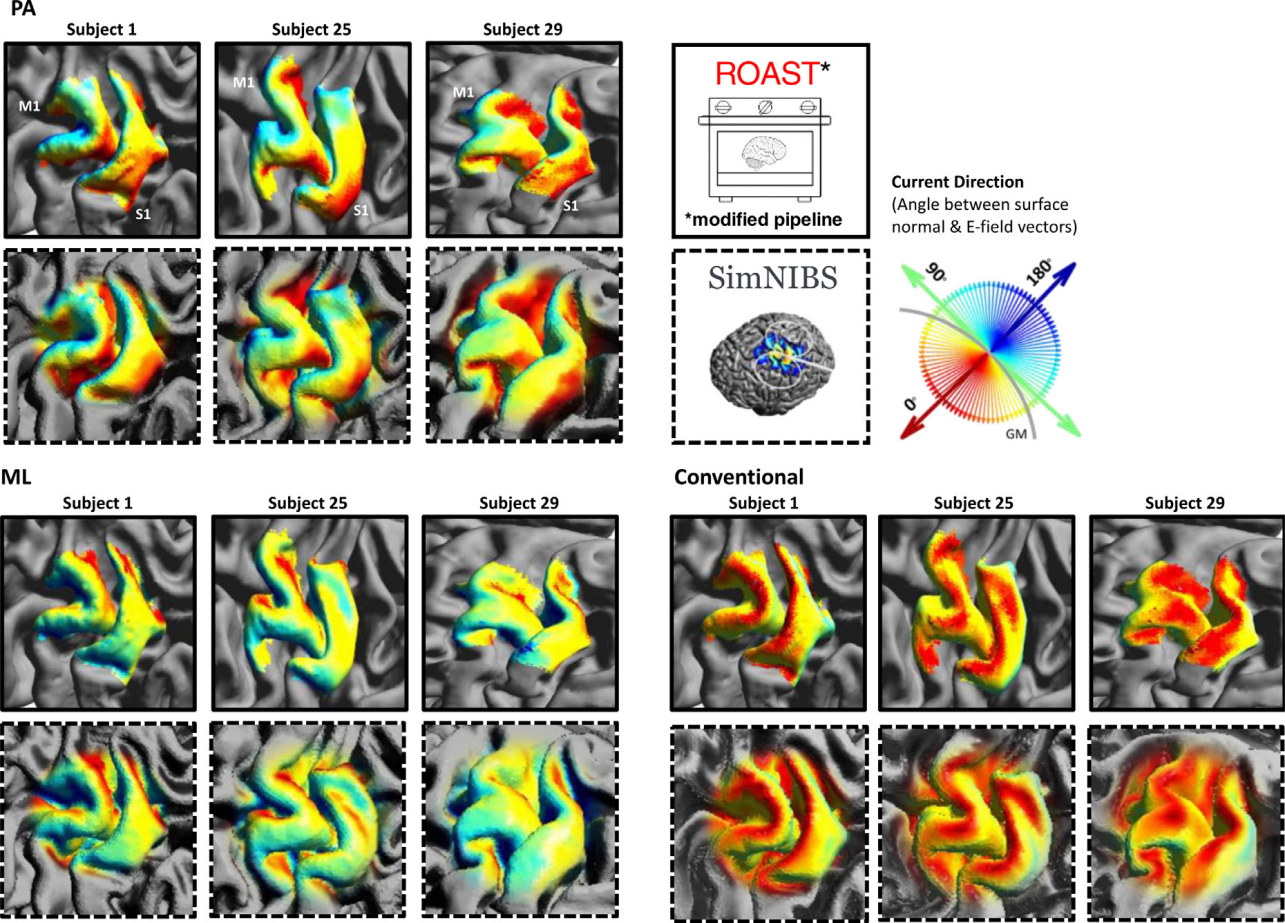
**Comparing modified ROAST and SimNIBS v3.2 pipelines: current direction for each montage in three subjects.** Current direction (angle in degrees between surface normal and E-field vectors) is depicted across the pre- and post-central gyri after running modified ROAST (solid boxes) and SimNIBS (dashed boxes) pipelines. SimNIBS produces angle in radians across the same colour spectrum: minimum angular difference (blue) rad = 0 (0°); midpoint (green) rad = 1.54 (88°), maximum (red) rad = 3.08(176°). Note the consistency in pattern of current direction across pipelines.
